# Chronic MPTP in Mice Damage-specific Neuronal Phenotypes within Dorsal Laminae of the Spinal Cord

**DOI:** 10.1007/s12640-020-00313-x

**Published:** 2020-11-18

**Authors:** Francesca Biagioni, Giorgio Vivacqua, Gloria Lazzeri, Rosangela Ferese, Simone Iannacone, Paolo Onori, Sergio Morini, Loredana D’Este, Francesco Fornai

**Affiliations:** 1 I.R.C.C.S. Neuromed, via dell’Elettronica, Pozzilli, Italy; 2 Integrated Research Center (PRAAB), Campus Biomedico University of Roma, Via Alvaro del Portillo 21, 00125 Roma, Italy; 3 Department of Anatomic, Histologic, Forensic and Locomotor Apparatus Sciences, Sapienza University of Roma, Via Alfonso Borelli 50, 00161 Roma, Italy; 4 Department of Translational Research and New Technologies in Medicine and Surgery, University of Pisa, Via Roma 55, 56126 Pisa, Italy

**Keywords:** Alpha-synuclein, Calbindin D28K, Calretinin, Enkephalins, Parkinson’s disease, Parvalbumin, Substance P

## Abstract

The neurotoxin 1-methyl, 4-phenyl, 1, 2, 3, 6-tetrahydropiridine (MPTP) is widely used to produce experimental parkinsonism. Such a disease is characterized by neuronal damage in multiple regions beyond the nigrostriatal pathway including the spinal cord. The neurotoxin MPTP damages spinal motor neurons. So far, in Parkinson’s disease (PD) patients alpha-synuclein aggregates are described in the dorsal horn of the spinal cord. Nonetheless, no experimental investigation was carried out to document whether MPTP affects the sensory compartment of the spinal cord. Thus, in the present study, we investigated whether chronic exposure to small doses of MPTP (5 mg/kg/X2, daily, for 21 days) produces any pathological effect within dorsal spinal cord. This mild neurotoxic protocol produces a damage only to nigrostriatal dopamine (DA) axon terminals with no decrease in DA nigral neurons assessed by quantitative stereology. In these experimental conditions we documented a decrease in enkephalin-, calretinin-, calbindin D28K-, and parvalbumin-positive neurons within lamina I and II and the outer lamina III. Met-Enkephalin and substance P positive fibers are reduced in laminae I and II of chronically MPTP-treated mice. In contrast, as reported in PD patients, alpha-synuclein is markedly increased within spared neurons and fibers of lamina I and II after MPTP exposure. This is the first evidence that experimental parkinsonism produces the loss of specific neurons of the dorsal spinal cord, which are likely to be involved in sensory transmission and in pain modulation providing an experimental correlate for sensory and pain alterations in PD.

## Introduction

Parkinson’s disease (PD) is the second-most prevalent neurodegenerative disease (Ascherio et al. 2016). PD-related neurodegeneration involves dopamine (DA) neurons of the substantia nigra pars compacta (SNpc) and noradrenaline (NA) neurons of the locus coeruleus (LC), with a severe loss of DA and NA within specific regions of the central nervous system (CNS) (Ehringer and Hornykiewicz 1960, 1998; Gesi et al. 2000; Zarow et al. 2003; Tong et al. 2006; Surmeier et al. 2017). Nevertheless, neurodegeneration may involve other areas of the CNS, including spinal cord (Vivacqua et al. 2011a; Del Tredici and Braak 2012; Ferrucci et al. 2013; Yeh et al. 2016). The involvement of the spinal cord was suggested to underlay specific non-motor symptoms of PD, such as pain, orthostatic hypotension, or urinary dysfunctions (Natale et al. 2013; Schapira et al. 2017; Poewe et al. 2017). Accordingly, neuropathological studies detected degeneration of both autonomic regions and dorsal laminae in the spinal cord of PD patients (Braak et al. 2007; Del Tredici and Braak 2012). Alterations of specific dorsal intra-spinal circuits and/or degeneration of descending pathways from the brainstem (Del Tredici and Braak 2012; Surmeier et al. 2017) to the dorsal laminae could contribute to the development of non-motor symptoms of PD.

Degeneration of spinal motor neurons occurs in experimental parkinsonism induced by MPTP or rotenone (Ferrucci et al. 2013; Vivacqua et al. 2012, 2020; Samantaray et al. 2007, 2008a, 2008b, 2015).

However, no experimental model so far was tested to reproduce PD pathology within the dorsal horn of the spinal cord. This is key to understand the pathophysiology of pain and sensory alterations occurring in PD. In fact, pain is one of the most common non-motor symptoms in course of PD and sometimes anticipates the movement disorder. Currently, pain in PD is associated with a peripheral neuropathy affecting small fibers, although PD patients suffering pain may not feature such a neuropathy. This point leaves open the issue whether the involvement of sensory neurons involved in gate control may be affected in PD. In fact, the occurrence of alpha-synuclein aggregates in the dorsal spinal cord of PD patients is reported (Del Tredici and Braak 2012). Here we investigate whether specific neuronal cell bodies placed within spinal circuits involved in sensory and painful transmission are altered in chronic MPTP induced-parkinsonism. The doses of MPTP selected in the present study consists of low (5 mg/kg) doses chronically administered (21 days) in order to produce a slight damage to the nigrostriatal pathway while sparing nigral DA neurons. These experimental conditions mimic the pre-motor stage of PD, where sensory alterations do already occur.

## Materials and Methods

### Animals and MPTP Treatment

Twenty-six C57Bl/6N mice, weighting 22–25 g (Charles River, Italy), have been used in the present study. All animal protocols were approved by the local Animal Care and Use Committee of the Sapienza University of Roma and followed the European guidelines for Animal Care. Adequate measures were taken to minimize animal pain and discomfort.

The mice were housed for one week, two per cage, at 12-h light/dark cycles and with free access to food and water. After 7 days, mice were divided into two groups of thirteen mice each and treated as follows: the first group was treated intra-peritoneally (i.p.) with MPTP hydrochloride (purchased from Sigma, Milan, Italy), at the dose of 6 mg/kg (corresponding to 5 mg/kg of MPTP), dissolved in saline. MPTP was administered for twice daily at 6-h interval, for 21 days (cumulative dose, 210 mg/kg of MPTP). The saline-treated group was treated with saline, using the same schedule of administration. After treatments, mice were housed for 1 week, two per cage, at the same environmental conditions. Mice were sacrificed under chloral hydrate anesthesia, i.p. (360 mg/kg) (Sigma, Milan, Italy). Eighteen animals were processed for immunohistochemical analysis (eight mice were processed for paraffin embedding, while ten mice were perfused trans-cardially with paraformaldehyde); the remaining eight animals were used for western blot analysis.

### Tissue Preparation

Eight spinal cords (four from MPTP-treated mice and four from saline) and their brains were dissected out and immediately placed into a solution composed of ethyl alcohol (60%), acetic acid (10%), and chloroform (30%) and used for immunohistochemical analysis. Twenty hours later, brains and spinal cords were placed into 70% ethanol until they were included in paraffin. The brains were cut at microtome (Leica Microsystem, RM2125, Milan, Italy) into 10-µm-thick coronal sections for striatal TH density assessment, whereas 20-μm-thick were cut for nigral tyrosine hydroxylase-positive (TH^+^) cell stereological count. The spinal cords treated in this way were used for immunohistochemical staining with antibodies giving more clear-cut results in paraffin embedded sections rather than free-floating sections. The thickness of the sections was chosen in accordance with the quantification procedures applied (densitometric analysis of fibers). Eight animals were used for western blotting procedure.

The remaining ten animals were perfused through the left cardiac ventricle with 0.01 M phosphate-buffered saline (PBS), pH 7.4, followed by ice-cold PFA fixing solution (4% paraformaldehyde in 0.1 M phosphate buffered), containing 0.35% of glutaraldehyde. The spinal cords were quickly removed from the vertebral columns and the skulls, respectively. Specimens were post-fixed with cold PFA alone, for 24–48 h and stored at 4 °C in phosphate-buffered (PB) containing 15% of sucrose.

### Immunohistochemistry

Paraffin embedded sections of cervical spinal cord (10 µm) were used for immunohistochemical analysis. These specimens were used for Calbindin D28K, Calretinin, alpha-synuclein (alpha-syn), and parvalbumin immunostaining. The sections were treated with normal horse serum for 1 h (10% in PBS). Then, they were incubated overnight with primary antibody (see Table 1) and then for 1 h with secondary biotin-coupled anti-mouse IgG (1:200; Vector Laboratories, Burlingame, USA). Negative control sections from brains and spinal cords were performed without primary antibodies.

**Table 1:** Primary and secondary antibodies used in the study

Antibody	Distributor	Catalog number	RRID	Concentration
Monoclonal mouse anti-TH	Sigma Aldrich, Milan, Italy	Cod. T1299	AB_477560	1:100
Biotinylated horse anti-mouse IgG (H+L)	Vector lab. Burlingame, CA, USA	Cod. BA-2000	AB_2313581	1:200
Polyclonal rabbit anti-Calretinin	Merck Millipore, Burlington, MA, USA	Cod. AB5054	AB_11212775	1:100
Monoclonal mouse anti Calbindin D28K – Calbindin Antibody [CB-955]	Abcam, Cambridge, UK	Cod. ab82812	AB_1658451	1:100
Polyclonal rabbit anti Met-Enkephalin	Sigma Aldrich, Milan, Italy	Cod. AB5026	AB_2449196	1: 20.000
Monoclonal rat anti Substantia P (SP), clone NC1	Sigma Aldrich, Milan, Italy	Cod. MAB356	AB_2390979	1:30.000
Polyclonal rabbit anti-parvalbumin antibody	Abcam, Cambridge, UK	Cod. ab11427	AB_298032	1:100
ANTI a-synuclein	Sigma Aldrich, Milan, Italy; BD biosciences, San Jose, CA, USA	1:100; 1:800	AB_10746104; AB_398107	1:100; 1:800
Biotinylated Rabbit anti-rat (H+L)	Vector lab. Burlingame, CA, USA	Cod. BA-4000	AB_2336206	1:2000
Biotinylated Goat anti-rabbit (H+L)	Merck Millipore, Burlington, MA, USA	Cod. 401393	AB_437797	1:2000

Cryo-sections of spinal cord with a thickness of 25 µm were used for immunohistochemical analysis on free-floating sections for Met-Enkephalin and substance P (SP). In order to inactivate the endogenous peroxidase activity, the sections of spinal cord were pre-treated for 20 min at room temperature (RT) with PBS containing 0.1% sodium azide and 0.5% H_2_O_2_. Sections were incubated following the protocol of the M.O.M. kit (mouse on mouse, Vector Laboratories, Burlingame, USA), which includes a blocking reagent to avoid non-specific staining of endogenous mouse IgG. Conversely, when polyclonal antibodies, rinsed in rabbit, were used, the sections were pre-incubated for 30 min, at RT, with a normal serum from the same source of secondary antibody, diluted 1:50 in PBS. These steps were followed by incubation with antibody-dependent experimental conditions (Table 1) and subsequently with specific secondary antibodies diluted 1:1000 (Table 1). Both primary and secondary antibodies were diluted in PBS, containing 1% of Bovine Serum Albumine (BSA) and 0.3% of Triton X-100. After ABC-peroxidase complex step (ABC elite, Vector Laboratories, Burlingame, USA), peroxidase activity was evidenced using a solution containing 0.04% of 3,3′-diaminobenzidine-tetrahydrochloride (DAB, Fluka, Buchs, Switzerland), 0.4% of nickel ammonium sulfate, and 0.003% of H_2_O_2_ in 0.5 M Tris-HCl buffer, pH 7.6, for 3 min at RT.

### Densitometric Analysis of TH Immunoreactivity in the Striatum

Striatal TH immunoreactivity was semi-quantified by measuring relative optical densities. The corpus striatum was sectioned for the entire extent obtaining 10 levels for each animal, each spaced 160 μm. Each section was analyzed by measuring optical density. Images were acquired at low magnification (× 2.5), and the analysis was performed by assessing the intensity of the background values (i.e., the optical density measured in unlabelled areas present in the section, corpus callosum) by using Zeiss Axio Imager M1 microscope equipped with a motorized stage and focus control system (Zeta axis), and with a digital video camera. Results are expressed as mean ± S.E.M. for each group. Two sample *T* test (H_0_: *µ*_1_ = *µ*_2_) was used for statistical comparison of collected data. Hypothesis H_0_ was rejected when *p* < 0.05.

### Densitometric Analysis of Met-Enkephalin and SP Positive Fibers in the Dorsal Laminae of the Spinal cord

The immunostaining optical density for Met-enkephalin and SP were calculated, in cervical spinal cord, using a dedicated software (IAS, 2000, Delta Sistemi, Italy). Data analysis was performed in comparable cryo-sections of 25 μm each, acquired at low magnification (× 4.0) from the same level of the spinal cord, morphologically identified according to the mouse brain atlas of Paxinos and Franklin (2004). The cervical spinal cord was sectioned for entire, obtaining 40 levels for each animal, spaced 80 μm between them and lying within a total cervical cord length of around 8.0 mm. Data, referred to one side of each section examined, were submitted to statistical analysis. Results are expressed as the mean ± S.E.M. from each group. Two sample *T* test (H_0_: *µ*_1_ = *µ*_2_) was used for statistical comparison of collected data. Hypothesis H_0_ was rejected when *p* < 0.05.

### Stereological Count of Nigral TH^+^ Cells

The number of TH^+^ cells in the SNpc was assessed by stereological technique by using an optical fractionator, and a Zeiss Axio Imager M1 microscope equipped with a motorized stage and focus control system (Zeta axis), and with a digital video camera. The software Image-Pro Plus 6.2 for Windows (Media Cybernetics, Inc., Bethesda, MD) equipped with a Macro was used for the analysis of digital images. The Macro was obtained by “Immagine and Computer” (Bareggio, Italy) as already published (King et al. 2002).

The optical fractionator technique (adapted to 20-μm-thick sections) was used to count total cell number within SNpc (West et al. 1991; Gundersen et al. 1999). A systematically sampled series of sections every 160 μm (eight slices for each animal), spanning the entire extent of nigral formation, was selected for quantification. In each stained section, the area was identified and outlined at × 2.5 magnification. After outlining the regions of interest, a sampling grid of known dimensions (50 × 50 μm) was positioned over each area, and counting was carried out using a × 100 oil immersion lens (King et al. 2002).

### Western Blot Analysis

Four mice from each group were used for Western blot analysis for alpha synuclein (alpha-syn), calretinin and calbindin D28K. After sacrifice, the spinal cords were removed; the posterior horn was micro-dissected from the anterior horn (by using stereo-microscope for micro-dissection and considering the central canal as the reference point). Samples were homogenized in a lysis buffer containing 10 mM Tris–HCl, pH 7.4, 150 mM NaCl, 5 mM EDTA, 1% NP-40, 0.5% Na-deoxycholate, and 0.1% SDS. Twenty micrograms of proteins were separated by Mini-Protean TGX Precast gel gradient 4–20% (Biorad Laboratories, Hercules, CA, U.S.A), blotted by using Trans-Blot Turbo transfer pack 0.2-µm PVDF and reacted with primary antibody overnight 4 °C (Table 1). Horseradish peroxidase–conjugated goat-anti-mouse or goat-anti-rabbit IgG (1:3000) was used as secondary antibody (1 h RT), and immune-reactive bands were visualized by enhanced chemiluminescence (ECL Luminata Crescendo. EMD Millipore, Burlington, MA, USA) by using Chemidoc imaging system.

### Statistical Analysis

For densitometric analysis of SDS immune-blotting data are reported as the mean ± S.E.M. of 4 mice per group. Densitometry of striatal TH immunostaining, spinal Met-Enk, and SP immunostaining in dorsal laminae data are reported as the mean ± S.E.M. of 4 mice per group. For stereology the total number of TH^+^ neurons for each rostro-caudal level was computed from the formula: *N* = Σ (*n*) × 1/SSF × 1/ASF × 1/TSF) where *n* is the total number of neurons counted on each dissector, “SSF” (fraction of sections sampled) is the number of regularly spaced sections used for counts divided by the total number of sections through the entire extent of SN (= 1/5), “ASF” (area sampling frequency) is the dissector area divided by the area between dissectors (= 5625 μm^2^ × dissectors number)/region area), and “TSF” (thickness sampling frequency) is the dissector thickness divided by the section thickness. The total number of TH^+^ neurons in SNpc is the sum of the total number of neurons per each rostro-caudal level: *N* tot = Σ (Ni).

Total nigral TH^+^ cells are reported as the mean ± S.E.M. of 4 mice per group. For each method statistical comparison between groups was carried out using ANOVA with Scheffé’s test. Null Hypothesis H_0_ was rejected when *p* ≤ 0.05.

## Results

### Chronic Exposure to MPTP Reduces TH Immunostaining in the Dorsal Striatum, While not Reducing Nigral TH^+^ Cell Bodies

**Fig. 1:**
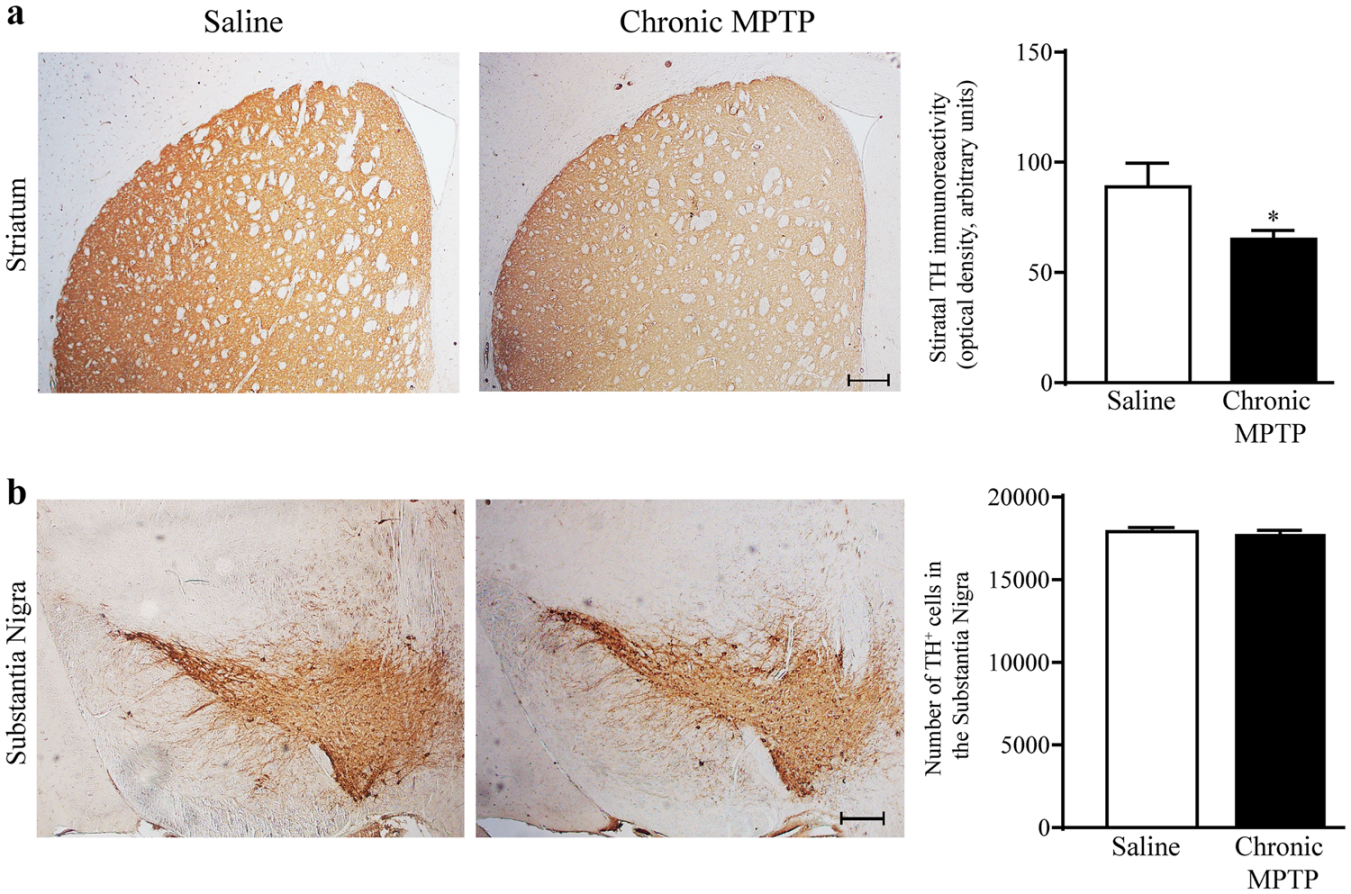
Chronic exposure to MPTP decreases striatal TH immunostaining while preserving nigral TH immune-positive cell bodies. **a** Representative pictures from TH immune-stained striatum following chronic exposure to low amounts of MPTP for 21 consecutive days (cumulative dose 210 mg/kg of free MPTP). A slight loss of TH immune-positive terminals in the dorsal striatum is present (Fig. 1a—scale bar 200 µm) as reported in the graph measuring optical density. **b** Representative pictures from TH immune-stained substantia nigra where no neuronal loss is present as measured in the graph reporting stereological counts. Results are expressed as the mean ± S.E.M. from 4 mice per group. Null hypothesis (H_0_) was rejected when *P* ≤ 0.05. Scale bar = 200 µm

Chronic exposure to low amounts of MPTP (cumulative dose of 210 mg/kg) reduces the TH immunostaining within the dorsal striatum of MPTP-treated mice compared with saline-treated mice (*n* = 4 each group). Densitometric analysis of TH^+^ terminals confirms the occurrence of a slight (roughly 20%) decrease of TH immunostaining within the dorsal striatum of MPTP-treated mice, compared with saline-treated mice (Fig. 1a). No effect in the number of TH^+^ cell bodies was observed within the SNpc (*n* = 4 each group), as shown in the representative pictures of Fig. 1b. The count of TH^+^ cell bodies within the SNpc provided by stereology count demonstrates that the number of nigral TH^+^ neurons is not affected by this protocol of chronic MPTP exposure (Fig. 1b). This markedly differs from continuous MPTP exposure (Fornai et al. [Bibr CR23][Bibr CR23], b; Jackson-Lewis and Przedborski 2007; Gibrat et al. 2009) or chronic MPTP + probenecid (Petroske et al. 2001), as well as from acute injection of MPTP as we have previously reported (Vivacqua et al. 2012).

### Loss of Calbindin D28K, Calretinin, and Parvalbumin Neurons and Fibers in the Dorsal Laminae of Spinal Cord After Chronic Exposure to MPTP

**Fig. 2:**
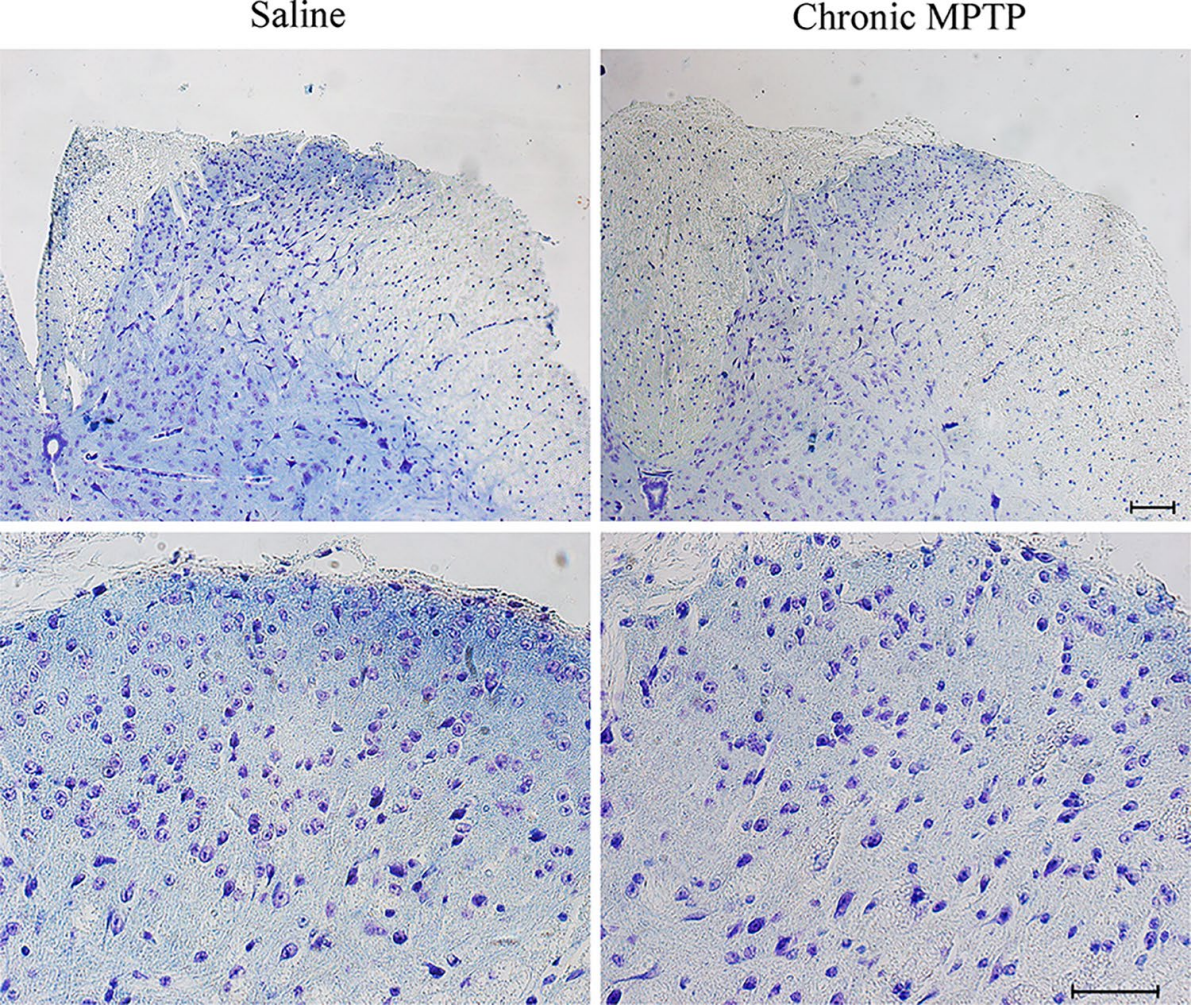
Nissl staining of dorsal horn. Representative pictures from Nissl staining shows a decrease in the cellular density within the dorsal horn following exposure to chronic MPTP. Upper lane low and Lower lane high magnification. Scale bar = 100 µm and 50 µm, respectively

**Fig. 3:**
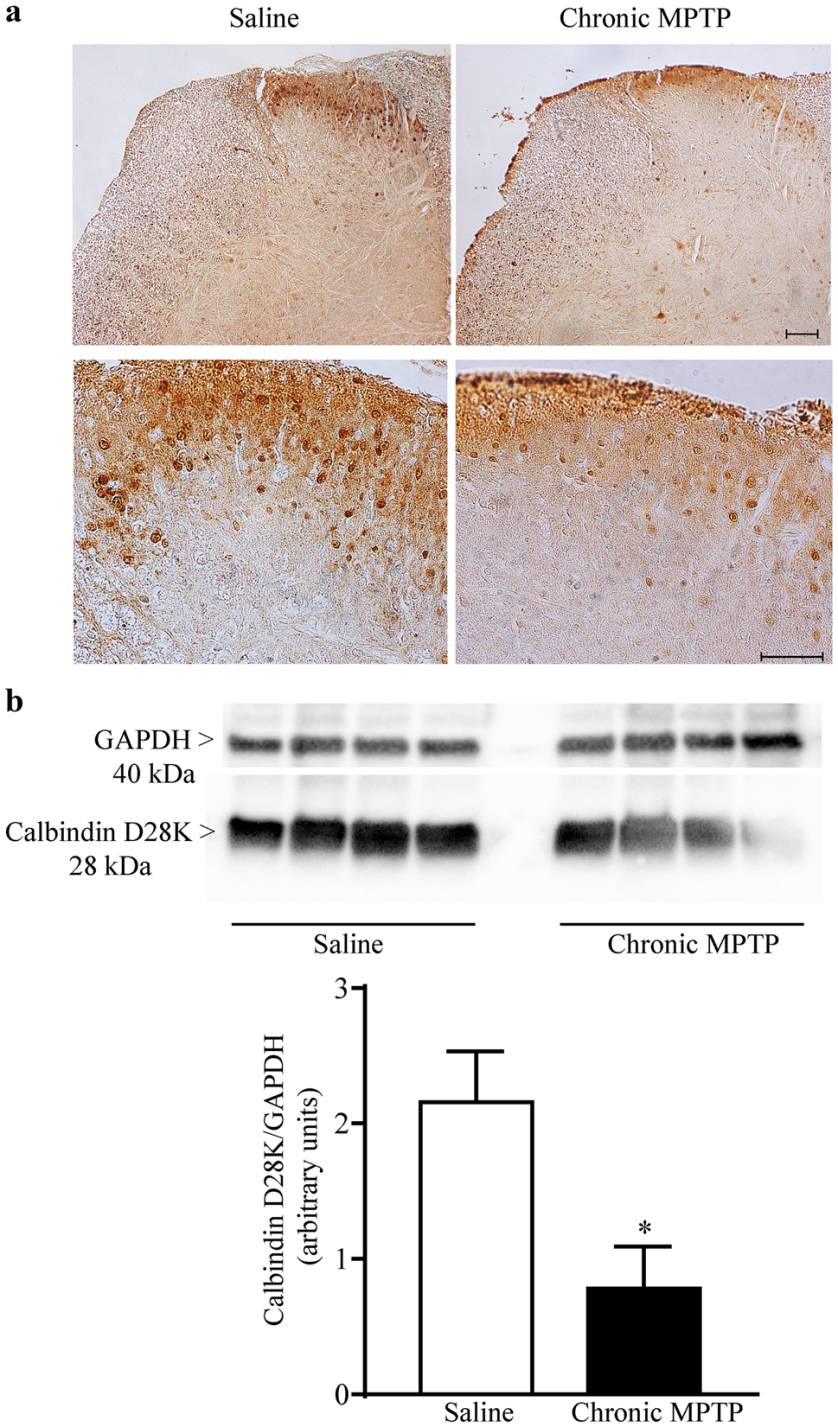
Calbindin D28K immunostaining. **a** Representative pictures from calbindin D28K immune-stained dorsal horn and **b** Western-blot analysis for calbindin D28K. Chronic MPTP decreases calbindin D28K immunostaining in the dorsal horn as shown in representative pictures at low (upper lane) and high magnification (lower lane). This is confirmed by western blots which are quantified in the graph reporting optical density. Results are expressed as the mean ± S.E.M. from 4 mice per group. Null hypothesis (H_0_) was rejected when *P* ≤ 0.05. Scale bar = 100 µm and 50 µm (low and high magnification, respectively)

**Fig. 4:**
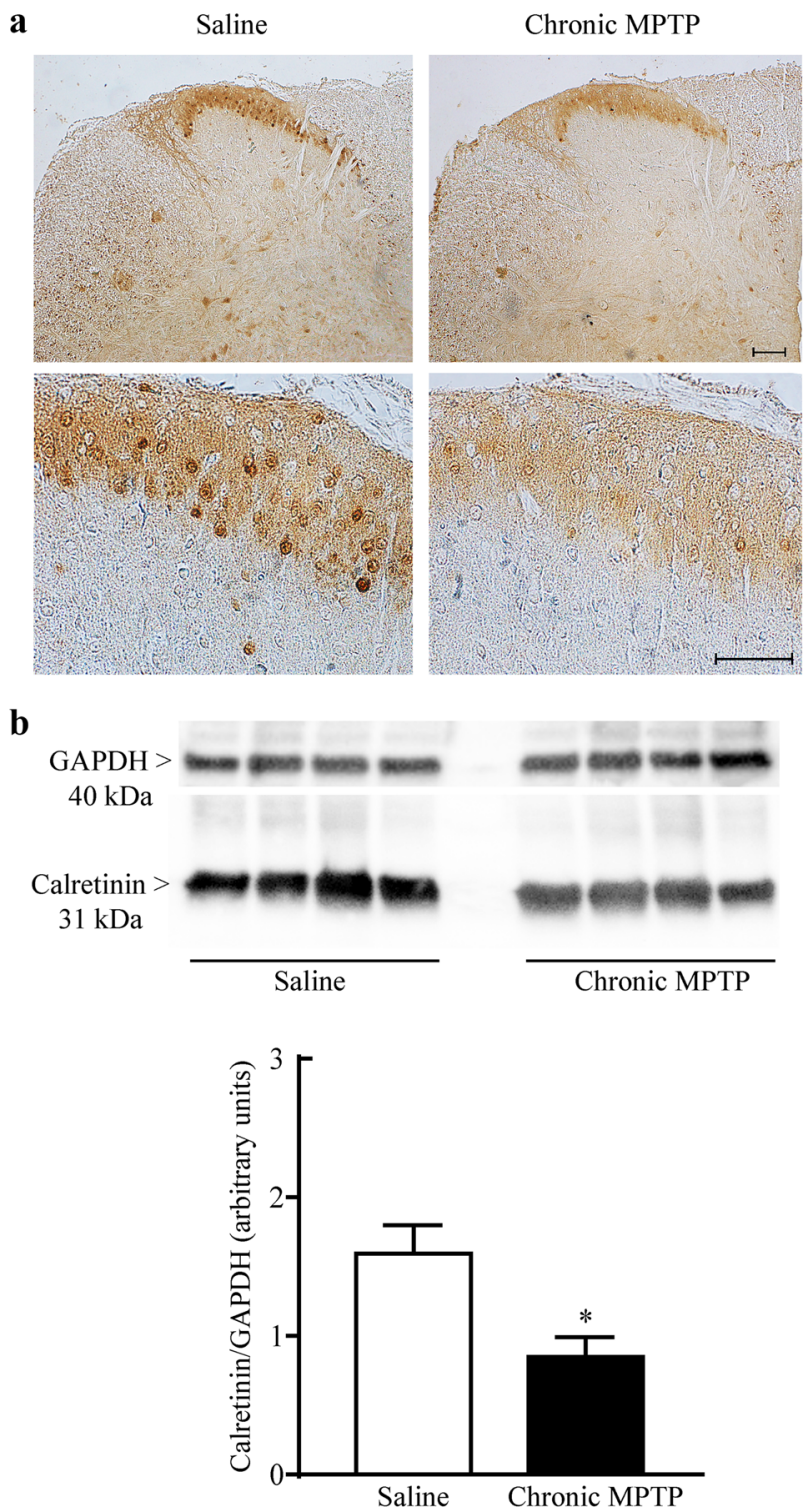
Calretinin immunostaining. **a** Representative pictures from calretinin immune-stained dorsal horn and **b** Western-blot analysis for calretinin. Chronic MPTP decreases calretinin immunostaining in the dorsal horn as shown in representative pictures at low (upper lane) and high magnification (lower lane). This is confirmed by western blots which are quantified in the graph reporting optical density. Results are expressed as the mean ± S.E.M. from 4 mice per group. Null hypothesis (H_0_) was rejected when *P* ≤ 0.05. Scale bar = 100 µm and 50 µm (low and high magnification, respectively)

**Fig. 5:**
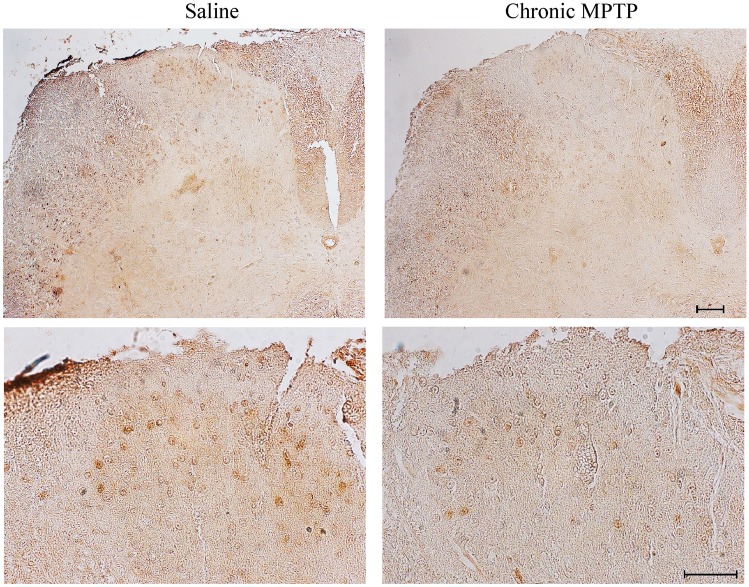
Parvalbumin immunostaining. Representative pictures from calretinin immune-stained dorsal horn. Chronic MPTP decreases parvalbumin immunostaining in the dorsal horn as shown in representative pictures at low (upper lane) and high magnification (lower lane). Scale bar = 100 µm and 50 µm (low and high magnification, respectively)

An overall decrease in cellular density has been detected in the posterior horn of the spinal cord of MPTP-treated mice comparing with saline-treated mice, as demonstrated by Nissl staining (Fig. 2). calbindin D28K (Fig. 3), calretinin (Fig. 4), and parvalbumin (Fig. 5) identify populations of dorsal horn neurons which are involved in sensory processing including pain modulation. Immunohistochemistry and western blot analysis have been carried out. Chronic MPTP produced a severe loss of calbindin D28K, calretinin, and parvalbumin immune positive neurons in the dorsal laminae of the spinal cord. An overall loss of calbindin D28K (Fig. 3a), calretinin (Fig. 4a), and parvalbumin (Fig. 5) positive neurons occurs in laminae I, II, and III of MPTP-treated mice. Western blot analysis confirms reduced levels of calbindin D28K (Fig. 3b) and calretinin (Fig. 4b) in the homogenates from micro-dissected dorsal spinal cord (*p* < 0.05).

In saline-treated mice, slightly stained neurons, immune-reactive for parvalbumin, were detected in the inner lamina II, at the border with lamina III (Fig. 5 - left panel, high magnification). These neurons are also lost after chronic MPTP-exposure, and spared neurons are characterized by pale bodies with a faint parvalbumin immunostaining (Fig. 5 - right panel, high magnification).

### Met-Enkephalin and Substance P Immunoreactivity in the Dorsal Spinal Cord from MPTP-Treated Mice

**Fig 6:**
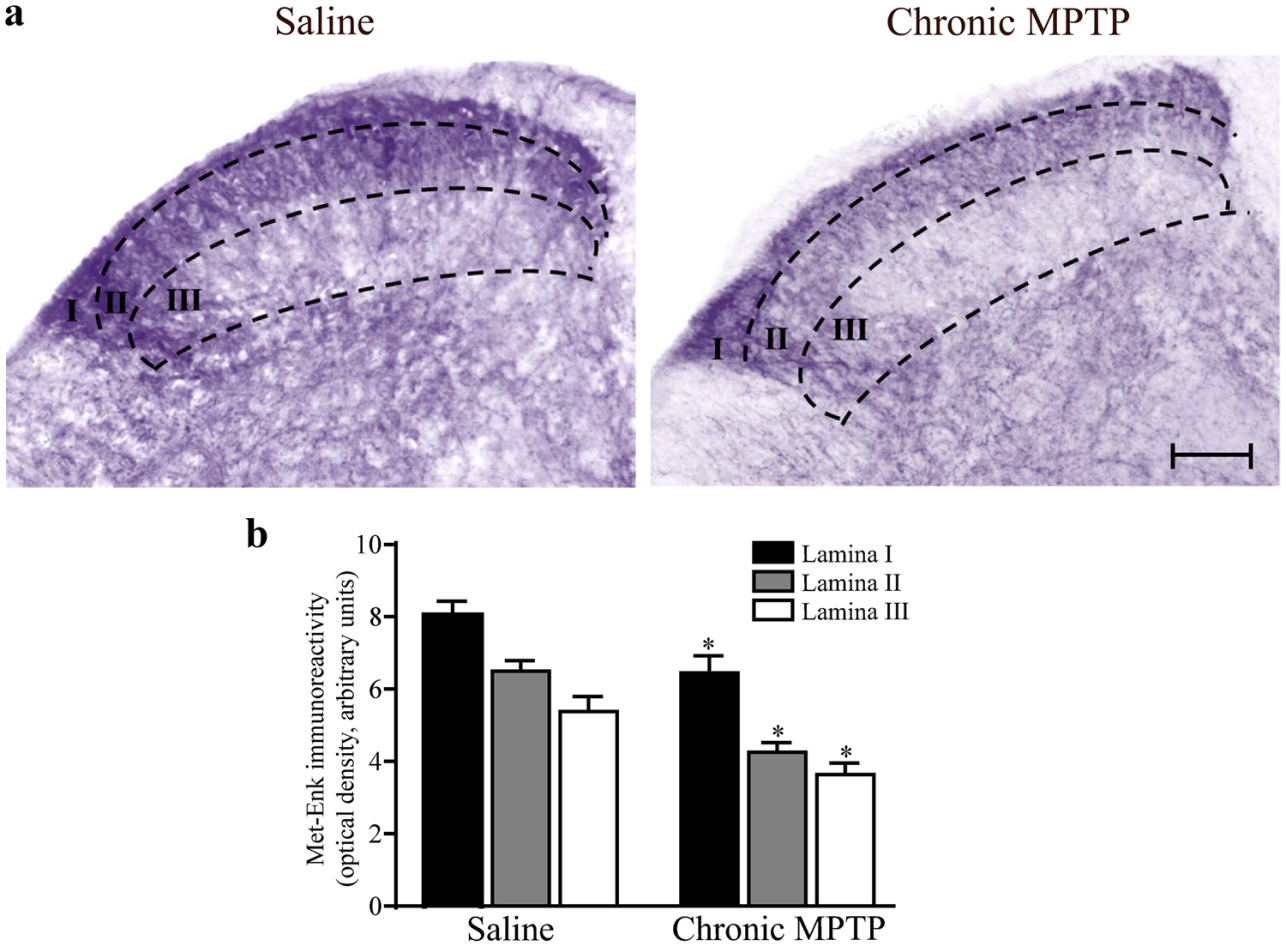
Met-Enkephalin immunostaining. **a** Representative pictures from Met-Enkephalin immune-stained dorsal horn and **b** optical densitometry from Met-Enkephalin immune-stained slices from dorsal horn (laminae I; II; III). Chronic MPTP decreases Met-Enkephalin immunostaining in the dorsal horn as shown in representative pictures. This is confirmed by optical densitometry reported in the graph for each lamina. Results are expressed as the mean ± S.E.M. from 5 mice per group. Null hypothesis (H_0_) was rejected when *P* ≤ 0.05. Scale bar = 60 µm

**Fig. 7:**
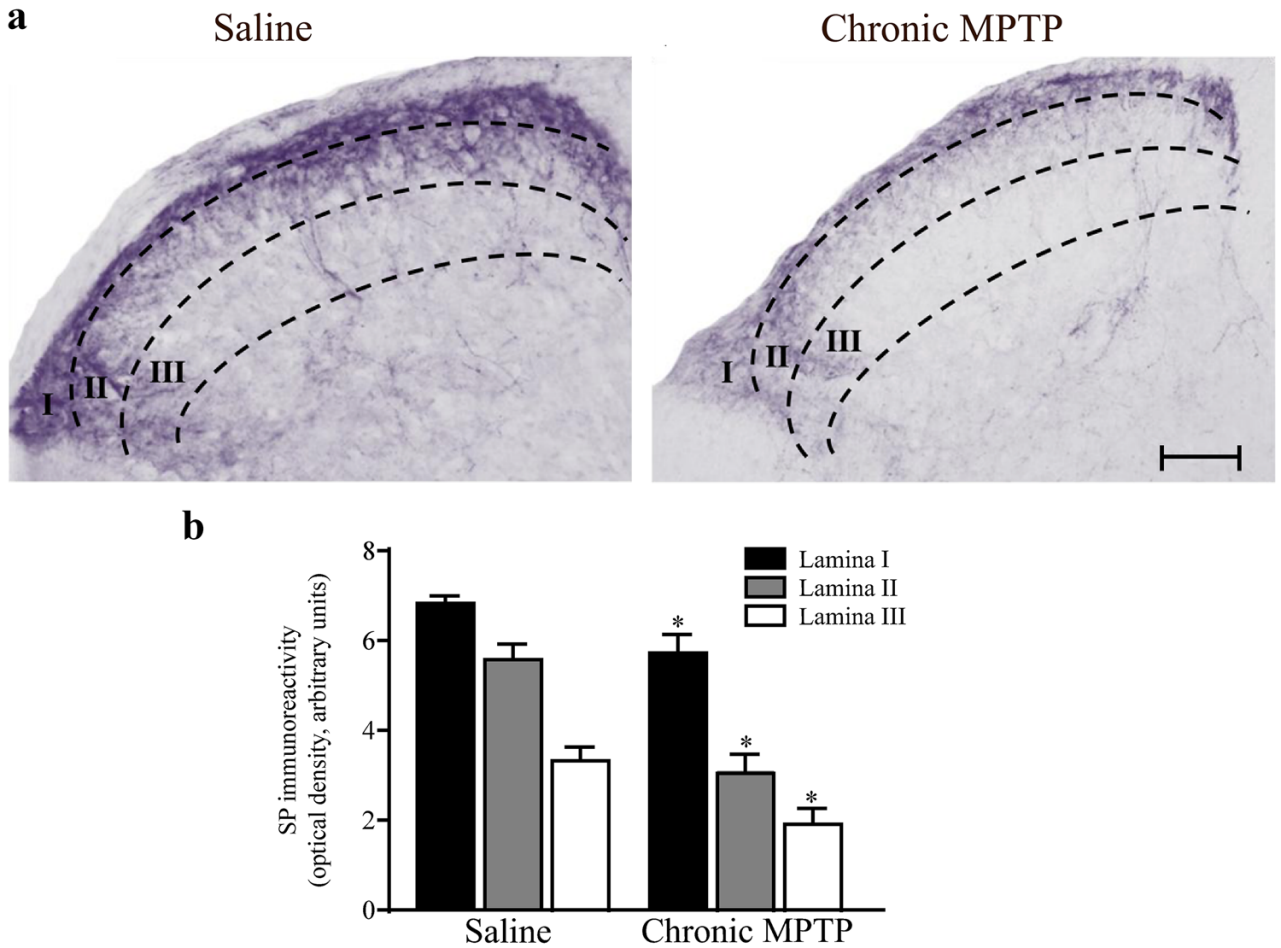
Substance P immunostaining. **a** Representative pictures from SP immune-stained dorsal horn and **b** Optical densitometry from SP immune-stained slices from dorsal horn (laminae I; II; III). Chronic MPTP decreases SP immunostaining in the dorsal horn as shown in representative pictures. This is confirmed by optical densitometry reported in the graph for each lamina. Results are expressed as the mean ± S.E.M. from 5 mice per group. Null hypothesis (H_0_) was rejected when *P* ≤ 0.05. Scale bar = 60 µm

In saline-treated mice, packed Met-Enk immune-reactive fibers and neurons were detected in laminae I and II, whereas a widespread network of thin Met-Enk fibers and neurons were observed within lamina III and in the dorsal part of lamina IV (Fig. 6a). Similarly, intense immunoreactivity for SP was present in laminae I and II, while only faint and sparse SP immunostaining occurs in lamina III and in the dorsal part of lamina IV (Fig. 7a), suggesting a wide distribution of SP immune-reactive fibers throughout the posterior spinal cord.

Chronic MPTP produces severe loss of Met-Enk and SP immune-reactive fibers in both laminae I and II (Figs. 6b and 7b), along with reduction of immunostaining for Met-Enk and SP detectable in lamina III and in the dorsal part of lamina IV (Figs. 6b and 7b).

Reduction of SP and Met-Enk immunoreactivity, following MPTP, was semi-quantified by densitometry for both neuropeptides in laminae I, II, and III following chronic MPTP. The decrease was prevalent within laminae II and III (Figs. 6 and 7 - right panel).

### Alteration of Alpha-Syn Immunostaining in the Dorsal Laminae of the Spinal Cord after Chronic MPTP Exposure

**Fig. 8:**
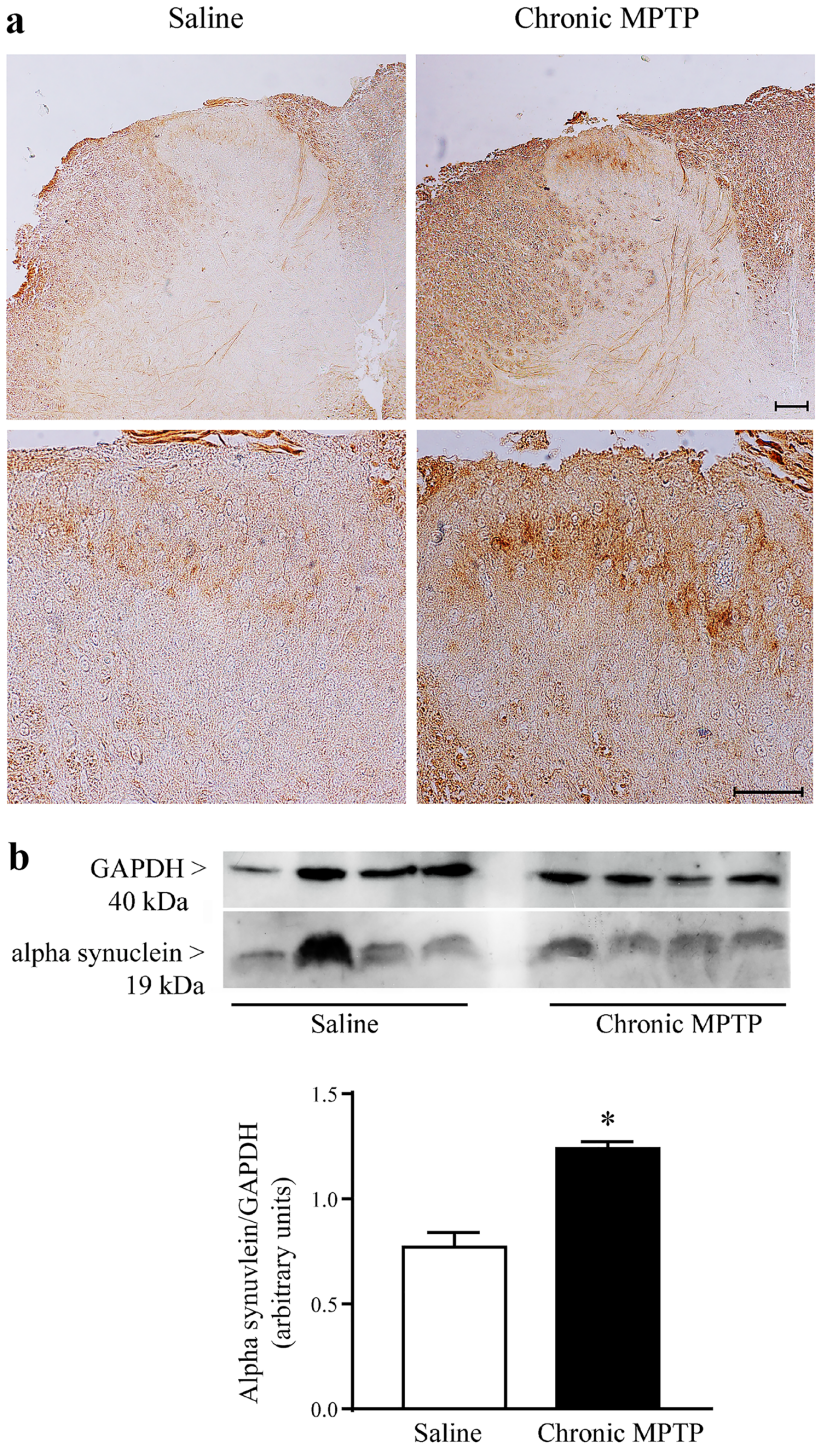
alpha-synuclein immunostaining. **a** Representative pictures from alpha-syn immune-stained dorsal horn and **b** Western-blot analysis for alpha-syn. Chronic MPTP increases alpha-syn immunostaining in the dorsal horn as shown in representative pictures at low (upper lane) and high magnification (lower lane). This is confirmed by western blots which are quantified in the graph reporting optical density. Results are expressed as the mean ± S.E.M. from 4 mice per group. Null hypothesis (H_0_) was rejected when *P* ≤ 0.05. Scale bar = 100 µm and 50 µm (low and high magnification, respectively).

Alpha-syn immunostaining is the culprit of neurodegeneration in PD. In previous studies, alpha-syn overexpression has been reported in spinal motor neurons, after acute and chronic exposure to MPTP (Vivacqua et al. 2012, 2020). In the present study alpha-syn immunohistochemistry was detected in the dorsal horn. In saline-treated mice alpha-syn immunostaining was faint and scattered within laminae I and II (Fig. 8a). Following MPTP, an overall increase in alpha-syn immunostaining was detected (Fig. 8a). Alpha-syn accumulates appears mostly within lamina II neurons (Fig. 8a).

Western-blot analysis for alpha-syn confirms such an increase within homogenates of micro-dissected dorsal horn from MPTP-treated mice (Fig. 8b).

## Discussion

In the present study we report that chronic exposure to low amounts of the neurotoxin MPTP, which does not decrease DA nigral cell bodies, induces severe neurotoxicity within the dorsal laminae of the spinal cord. This effect was obtained by the chronic administration of MPTP at doses, which do not affect severely the basal ganglia, which suggests a lower threshold for dorsal spinal cord compared with nigral damage.

This is similar to the threshold for motor neuron damage we recently published (Vivacqua et al. 2020). In the dorsal horn a severe neuronal loss occurs in the most dorsal laminae within the dorsal horn itself. The neuronal phenotypes being affected by MPTP administration correspond to those typical of laminae I-III (calbindin D28K, calretinin parvalbumin enkephalins positive neurons). Such a loss was confirmed by a marked decrease of these antigens at western blotting. The loss of specific sensory neurons being involved in nociception was associated with a marked increase of alpha-syn immunostaining within the same area of the dorsal horn.

MPTP toxicity consists in inhibiting the mitochondrial complex I respiratory chain and inducing oxidative stress and ATP depletion in target neurons. MPTP neurotoxicity is due to the oxidation of MPTP to its drawn neurotoxin 1-methyl, 4-phenyl pyridinium ion (MPP^+^) operated by the monoamine oxidase type B (MAO B) within astrocytes. MPP^+^ is an ion which is specifically transported within neurons through dopamine and norepinephrine transporters (DAT and NET). These transporters are present indeed within neurons belonging to the dorsal laminae of the spinal cord (Samantaray et al. 2008a, b; Aira et al. 2016). Similarly, MAO B is present within astrocytes of the spinal cord (Chiba et al. 1984; Markey et al. 1984).

Therefore, neuronal loss in the dorsal laminae could be due to a direct action of MPP^+^ within the cells. The inhibition of mitochondrial respiratory chain, operated by MPP^+^, is expected to increase the production of oxygen free radicals within the mitochondria, resulting in lipoperoxidation and rupture of mitochondrial membranes, release of cytochrome C, and activation of the intrinsic apoptotic cascade. Apoptosis of target cells, in turn, leads the degeneration of neuronal axons, thus explaining the wide loss of fibres detectable in the spinal cord of MPTP-treated mice.

On the other hand, repetitive low-level mitochondrial damage, produced by chronic MPTP administration, may induce alpha-syn aggregation in dorsal spinal neurons, probably through the inhibition of the ubiquitin-proteasome system and autophagy (Fornai et al. 2005a, 2005b; Ferrucci et al. 2008) or through direct modification of alpha-syn conformation and alpha-syn-lipids interaction. Alpha-syn aggregation, in turn, elicits mitochondrial dysfunction (Ludtmann et al. 2018; Martínez et al. 2018), further fuelling this vicious circle.

Increased alpha-syn immunostaining detected in the dorsal laminae of MPTP-treated mice has been substantiated by using both immunohistochemistry and western blot. We found that, following chronic MPTP administration, alpha-syn immunostaining was markedly altered in laminae I, II and III. These findings mimic the data obtained by Del Tredici and Braak in the spinal cord from PD patients (Del Tredici and Braak 2012).

These neurons are involved in nociceptive transmission and modulation, and interestingly, as previously reported by Braak and colleagues (Del Tredici and Braak 2012), nociceptive neurons and interneurons of laminae I and II chiefly displayed alpha-syn pathology in post-mortem specimens from PD patients. This is consistent with alpha-syn pathology affecting nociceptive amyelinic fibres early in PD (Braak et al. 2001), which were reported to accumulate alpha-syn in both humans and rodents (Vivacqua et al. 2009; 2011b). Moreover, these axons are sensitive to alteration of microtubules dynamics (Colvin et al. 2019). Hence, they easily succumb to axonal transport defects, like those occurring during mitochondrial dysfunction (Abou-Sleiman et al. 2006) in the course of synucleinopathies.

Chronic pain is a common symptom in the course of PD, and about 60–70% of PD patients experience various type of pain including musculoskeletal and visceral. Pain represents is an early symptom in PD, since it often precedes motor symptoms. Notwithstanding the clinical relevance, very few studies have investigated so far the sensory neurons in the experimental models of parkinsonism. Park and colleagues (2015) have shown that injection of MPTP 4 × 20 mg/kg body weight at 2-h interval, resulted in a drop of paw withdrawal thresholds upon thermal (tail flick) and mechanical (von Frey) stimulation. However, they did not investigate neuronal mechanism underlying these clinical features. Indeed, neither genetic nor toxic experimental models of parkinsonism have previously reproduced the lesion of pain related neuronal circuits.

From a clinical point of view, pain in PD has been related to peripheral sensory neuropathy or to fluctuations in Levo-DOPA therapy and patients suffering from PD pain have been subdivided clinically into those with or without sensory neuropathy (Lin et al. 2016; Nandhagopal et al. 2010; Nolano et al. 2008). Peripheral sensory neuropathy has been linked to polyneuropathic pain but also to musculoskeletal or widespread pain (Berglund et al. 2002; Kosek et al. 1996; Uceyler et al. 2018). Here we report for the first time that the chronic exposure to the parkinsonism-inducing neurotoxin MPTP leads a profound degeneration of sensory neurons likely involved to pain modulation.

In detail, the markers we used provide a reliable identification of specific classes of neurons and fibers within the sensory pathways of the dorsal spinal cord. In detail, calbindin D28K marks quite specifically those neurons of lamina I and dorsal part of lamina II (Gutierrez-Mecinas et al. 2018), which are involved in the nociceptive projection to the lateral cervical nucleus via the spinocervical tract (Ren and Ruda 1994). These neurons are also involved in the projection of painful stimuli along the anterolateral pathways. In any case, these neurons are key in carrying sensory information and provide a seminal component for gate control (Melzack and Wall 1962;1965; Eccles et al. 1960; 1961a;b; 1962). Calretinin possesses a topographical arrangement, which overlaps with calbindin D28K and substance P. Specifically, these neurons of lamina I and dorsal lamina II are believed to provide for 30% of poly-segmental ascending pathways in the cord (Zhang et al. 2016; Gutierrez-Mecinas et al. 2018). Concerning Met-Enkephalin-containing neurons, these are abundant in the whole lamina II and dorsal lamina III sending their axons dorsally into lamina I and ventrally towards lamina IV. Met-Enkephalin staining merges with parvalbumin to identify lamina II and dorsal lamina III neurons which constitute the substantia gelatinosa. These neurons are involved to enhance the gate control by providing inhibition of pain-related small sensory fibers, which carry nociceptive information. The loss of these neurons is likely to reduce the threshold for pain as commonly observed in Parkinsonian patients (Ribeiro-da-Silva and Coimbra 1982; Ribeiro-da-Silva et al. 1991; Zhang et al. 2016).

Calretinin interneurons are the largest population in laminae I and II of the spinal cord (Ren and Ruda 1994; Gutierrez-Macinas et al. 2019). A small part (25%) of these neurons co-express SP (Haring et al. 2018; Sathyamurthy et al. 2018). Activation of most calretinin positive (apart from those expressing substance P) cells is reported to cause a reduction of von Frey threshold and facilitation of transmission of mechanical stimuli (Duan et al. 2014; Peirs et al. 2015); a clinical picture reported also in MPTP models of parkinsonism (Park et al. 2015). In our study, we report an overall reduction of calretinin immune-reactive cells in the inner dorsal and in the outer layer of lamina II, concomitant with a significant reduction of SP immunoreactivity in the same lamina and in the outer part of lamina III. Whether these anatomical alterations contribute to the pathophysiology of pain is not clear. It is likely that a loss of these neurons (calretinin interneurons placed in the outer layer of lamina II) contribute with the loss of Met-Enkephalin-containing neurons to reduce pain threshold. A recent study has reported that calretinin immune-reactive interneurons form an interconnected network that can initiate and sustain enhanced excitatory signalling, and directly relay signals to lamina I projection neurons, being important for the generation and amplification of pain (Smith et al. 2019).

Calbindin D28K positive neurons are mainly present in lamina I and in the outer layer of lamina II. Calbindin is closely associated with calcium buffering, and it is specifically expressed by excitatory neurons (Antal et al. 1991). However, calbindin D28K positive interneurons of Laminae I, II, and III are morphologically and physiologically heterogeneous. Craig et al. (1994, 2002) have shown that calbindin D28K neurons of lamina I are mainly projecting neurons sending their axons in the anterolateral system to the ventro-posteromedial nucleus (VPM) of the thalamus, in response to pain and temperature. Calbindin neurons of laminae II and III, instead, are mainly considered as interneurons, involved in the modulation of pain and somatosensory inputs, through both excitatory and inhibitory activity, as determined by electrophysiological studies (Yasaka et al. 2010). Here an overall loss of calbindin D28K positive neurons in laminae I, II, and III was documented. As for calretinin, contribution of different subtypes of calbindin D28K positive neurons should be investigated by neurophysiological studies.

Following MPTP a profound loss of parvalbumin positive neurons occurs at the border between lamina II and lamina III. Parvalbumin positive neurons are inhibitory GABAergic or glycinergic interneurons, the cell bodies is placed within lamina III, and their axons move dorsally to internal-ventral layer of lamina II, taking part to a complex network of interneurons which modulate the vertical glutamatergic cells of the outer layer of lamina II. These neurons are supposed to inhibit projecting sensory neurons of lamina I. Loss of these inhibitory interneurons could activate pain transmission. Parvalbumin interneurons are activated by the myelinic Aβ fibres transmitting mechanical stimuli. Such an activation, during light touch, inhibits the engagement of nociceptive network. The loss of these interneurons may explain the neurophysiological and behavioural features of mechanical allodynia reported by (Park et al. 2015) in MPTP-treated animals. This might also explain mechanical hypersensitivity reported in PD patients (Gierthmuhlen et al. 2009; Nolano et al. 2008).

The severe loss of Met-Enk and SP immunoreactivity in lamina II and III projecting in lamina I and IV is quite explicit. Met-Enk neurons are crucial for the modulation of pain transmission. They receive afferent nociceptive fibres from the periphery, and they are activated by descending serotoninergic and noradrenergic pathways from the brain stem. Activation of Met-Enk neurons leads the inhibition of projecting spino-thalamic neurons of lamina I as well the axo-axonic inhibition of fibres Aδ and C coming from the periphery and entering the spinal cord at laminae I and II. Disruption of Met-Enk network contributes to disinhibition of pain transmission and could represent a further mechanism underlying the development of chronic pain in experimental parkinsonism and PD. How SP positive interneurons loss can contribute to the development of chronic pain should be addressed by neurophysiological investigations; however, SP is also expressed by axons coming from the dorsal root ganglia and they involved in the transmission of pain and thermic inputs. The overall loss of SP we detected in lamina I is likely due also to a loss of small fibres. This mimics the loss of non-myelinic fibers occurring in PD patients (Nolano et al. 2008).

In conclusion, it is worth of attention that the predominant clinical phenotype in PD consists of a loss of thermal perception, abnormal heat pain, and mechanical hypersensitivity (Gierthmuhlen et al. 2009; Nolano et al. 2008). Although our study is carried out in MPTP mouse model, it shows the potential neuroanatomical correlates of sensory symptoms in PD patients. A loss of sensory peripheral fibres might contribute to a loss of thermal perception and occurrence of pathologic heat pain, whereas mechanical allodynia could be due to a paradoxical engagement of both spinocervical pathway and pain networks due to degeneration of projecting neurons and interneurons within laminae I, II, and II.
